# Modeling for Predictors of Knowledge Score on Etiology and Prevention Strategies for Cervical Cancer Among Women of Reproductive Age in Ibadan

**DOI:** 10.1200/GO.20.00086

**Published:** 2020-06-25

**Authors:** Imran O. Morhason-Bello, Yusuf Olushola Kareem, Isaac F. Adewole

**Affiliations:** ^1^ Department of Obstetrics and Gynecology, Faculty of Clinical Sciences, College of Medicine, University of Ibadan, Ibadan, Nigeria; ^2^ Institute of Advanced Medical Research and Training, College of Medicine, University of Ibadan, Nigeria; ^3^ Department of Epidemiology and Medical Statistics, Faculty of Public Health, College of Medicine, University of Ibadan, Ibadan, Nigeria

## Abstract

**PURPOSE:**

Poor knowledge regarding cervical cancer in at-risk populations directly affects health-seeking behavior and is associated with high mortality among women with cervical cancer. This study aims to evaluate the knowledge of women regarding the causes, risk factors, and prevention strategies of cervical cancer.

**METHODS:**

A multistage cross-sectional study of 1,002 women of reproductive age (18-49 years) in Ibadan was conducted. Knowledge of cervical cancer risk causes and prevention strategies was assessed using 13 and 9 question items, respectively. The knowledge score was graded as 0 (no knowledge), 1-4 (poor knowledge), or ≥ 5 (good knowledge). The proportional or partial proportional odds model was used to fit 3 models using the forward stepwise selection. All analysis was performed using Stata 15.0 (Stata Corp, College Station, TX).

**RESULTS:**

The median age of participants was 29 years (interquartile range [IQR], 23-35 years). The median knowledge scores of participants on causes and prevention strategies of cervical cancer were 3 (IQR, 0-4) and 3 (IQR, 0-5), respectively. The assessment of knowledge on causes and prevention strategies for cervical cancer revealed that having multiple sexual partners and no previous opportunity for counseling on cervical cancer screening were factors associated with lower odds of knowledge.

**CONCLUSION:**

The knowledge of women about the risk factors, causes, and prevention strategies of cervical cancer was poor. It is worrisome that poor knowledge was common among women with potential demographic risk factors for cervical cancer. We recommend innovative community mobilization to improve women’s knowledge of the risk factors associated with cervical cancer and prevention strategies.

## INTRODUCTION

Cervical cancer, the most common female genital tract cancer, is regarded as a disease related to sex and other inequalities. It is estimated that almost 9 of 10 of the 266,000 women who die of cervical cancer each year live in low- to middle-income countries.^1^ Africa, which has 16% of the global population, harbors 21% of new cervical cancers, and almost 1 in 4 African women diagnosed with cervical cancer die.^2,3^ These deaths can be prevented by well-organized public education on the risk factors associated with cervical cancer,^1,4^ vaccination, and early detection and treatment of premalignant diseases.

**CONTEXT**

**Key Objective**
We aimed to determine the knowledge level of women in a Nigerian community regarding cervical cancer causes, risk factors, and prevention strategies.
**Knowledge Generated**
A high proportion of women had poor knowledge scores regarding causes and risk factors (70.2%) and prevention strategies (72.6%) of cervical cancer. There was significant poor knowledge among women at high risk of developing cervical cancer.
**Relevance**
A context-specific community mobilization intervention targeting at-risk populations will help to improve knowledge and positive disposition toward the prevention and control of cervical cancer.

Generally, human papillomavirus (HPV) type 16 and 18 are the most common HPV types associated with cervical cancer worldwide.^5,6^ The evolution of the precancerous stage to cancer takes more than a decade.^3^ However, prevention and diagnostic programs are not widely available in countries with a low human development index, particularly in Africa and Asia.^1,7^ Cervical cancer is primarily prevented presently with 3 candidate HPV vaccines (Cervarix, GlaxoSmithKline Biologicals, Rixensart, Belgium; and Gardasil and Gardasil 9, Merck and Co, Inc., Whitehouse Station, NJ). HPV vaccination coverage is high in several Western countries but low in Africa and Asia.^8,9^

In Nigeria, cervical cancer is the second leading cause of cancer death after breast cancer in women.^10^ Nigeria recorded 14,943 new cervical cancers and 10,403 related deaths in 2018,^6^ accounting for 27.2% and 20.0% of all cancer diagnoses and cancer-related deaths, respectively, in the West Africa subregion. The majority of patients with cervical cancer present late in Nigeria, when definitive care is no longer feasible. In Nigeria, cervical cancer deaths increased from 8,240 in 2012 to 10,403 in 2018.^10,11^ Although Nigeria recently launched a new strategic policy to control cancer, including cervical cancer, implementation of this policy document has yet to be implemented nationwide. The HPV vaccines have not been included as part of national routine immunization, but these vaccines are administered on an out-of-pocket basis. Only a small percentage of the population has been vaccinated against HPV infection or has been screened for cervical cancer.^12,13^

The huge burden of cervical cancer in low- and middle-income countries has been associated with lack of public health information to prevent the disease, poor health-seeking behaviors, inadequate infrastructure for prevention strategies, lack of manpower to offer screening and early definitive treatment, and poor funding by the government.^7^ Many studies in Nigeria have shown mixed findings on the awareness of cervical cancer. The majority have reported a low level of awareness,^12,14,15^ whereas a few studies have reported a high level of awareness.^16,17^ The few studies that reported on the knowledge of cervical cancer in Nigeria showed that the knowledge was poor among women.^12,18^ However, most of these studies that reported on the knowledge of cervical cancer did not assess information on risk factors and primary and secondary prevention strategies. This study aims to evaluate the knowledge of women on risk factors and prevention strategies for cervical cancer.

## METHODS

This was a cross-sectional study; the data were extracted from the Human Papillomavirus Vaccine and Cervical Cancer Prevention Household Survey conducted within the Mokola community in Ibadan North Local Government Area. A total of 1,002 women of reproductive age (18-49 years) were recruited using multistage systematic sampling. Details about data collection, tools, and methods have been published elsewhere.^19^ Ethical approval was obtained from the University of Liverpool in the United Kingdom and Oyo State Ethical Review Committee, Ibadan, Nigeria.

### Data Management

#### Outcome variable.

The outcome variables were knowledge regarding causes of cervical cancer, which was evaluated using 13 questions, and knowledge about strategies to prevent cervical cancer, which was assessed using 9 questions. Each response was classified as yes, no, or not sure. A score of 1 was assigned for correct answers, whereas a score of 0 was assigned for wrong answers. The knowledge score on causes of cervical cancer was categorized as no knowledge (score, 0), poor knowledge (score, 1-4), or average knowledge (score, 5-6). A score of ≥ 7 was categorized as good knowledge. The knowledge scores regarding prevention of cervical cancer were categorized as no knowledge (score, 0), poor knowledge (score, 1-4), and good knowledge (score, ≥ 5).

#### Response variables.

There were 3 groups of response variables: sociodemographic variables, obstetric and sexual history, and exposure related to cervical cancer. The sociodemographic variables were age, occupation, marital status, family type, religion, ethnicity, income, and educational attainment. In this study, 200 naira (the official currency in Nigeria) were considered to equal 1 US dollar. The obstetric and sexual variables included number of pregnancies, number of living children, number of deliveries, number of living female children, ever had sex, age at first sex, and number of sexual partners. Variables related to cervical cancer that were included in the analysis were any experience of genital discharge or sores, any relative with cervical cancer, and any counseling regarding cervical cancer screening.

### Data Analysis and Management

The percentage distribution of response variables was computed. The Shapiro-Wilk test for normality was computed for continuous variables. Median, 25th and 75th percentile (interquartile range [IQR]) values were reported if *P* < .05. Kendall’s τ-b test of association between the outcome variables (knowledge about the causes and knowledge about prevention of cervical cancer) and the response variables was conducted. The Kruskal-Wallis test was used when the independent variables were nominal. Ordered logistic regression (proportional odds model) was then used to fit the 3 models using the forward stepwise selection procedure. However, a partial proportional odds model was fitted in situations where the parallel lines assumption for the use of proportional odds model was violated. The Brant test of parallel regression assumption was conducted, and an insignificant χ^2^ value suggests that the parallel lines assumption holds. The Akaike information criterion (AIC) and Bayesian information criterion (BIC) were used to assess a more parsimonious model when the parallel-lines constraint (ologit) and unconstraint (gologit) were imposed.^20,21^ The first model included all sociodemographic variables significant at *P* < .1. Next, each of the obstetric and sexual activity variables was adjusted in the second model, whereas history related to cervical cancer variables were adjusted in the third model. Only variables with a likelihood ratio χ^2^ of *P* < .1 were retained in the model. The findings in model 3 (full model) were used for the final interpretation of results at the *P* = .05 level of significance. A pairwise correlation matrix and variance inflation factor (> 5) were used as the cutoff to investigate multicollinearity between outcome and response variables.^22,23^ Number of pregnancies and number of deliveries were excluded as a result of collinearity. Participants with no responses and/or those with inconsistent responses for ever having had sex and age at first sex were excluded from the analysis. Responses were classified as inconsistent if respondents said they have never had sex but gave age at first sex and/or affirmed to have ≥ 1 sexual partner. All analyses were performed using Stata 15.0 software (Stata, College Station, TX).

## RESULTS

### Sociodemographic Characteristics, Information on Sexual Activity, and Exposure Related to Cervical Cancer of Respondents

The sociodemographic characteristics, sexual activity, and history related to cervical cancer among respondents are listed in Table 1. The median age of respondents was 29 years (IQR, 23-35 years). Most respondents were Yoruba (73.1%), Christians (76.0%), and in a monogamous relationship (79.0%) and resided with their partner (63.1%). Approximately 8 in 10 respondents had at least secondary education (84.2%), and more than half were semiskilled (56.2%), with a median income of 12,000 naira (IQR, 8,000-20,000 Naira). The majority of respondents were sexually active (84.1%) and had 1 partner (62.1%), and the median age at first sex was 20 years (range, 18-23 years). Three hundred forty-five respondents (34.4%) had heard of cervical cancer. Approximately 1 in 5 respondents had ever had genital discharge. Only 3% had a relative with cervical cancer, and 4% reported ever being counseled for cervical cancer screening.

**TABLE 1:**
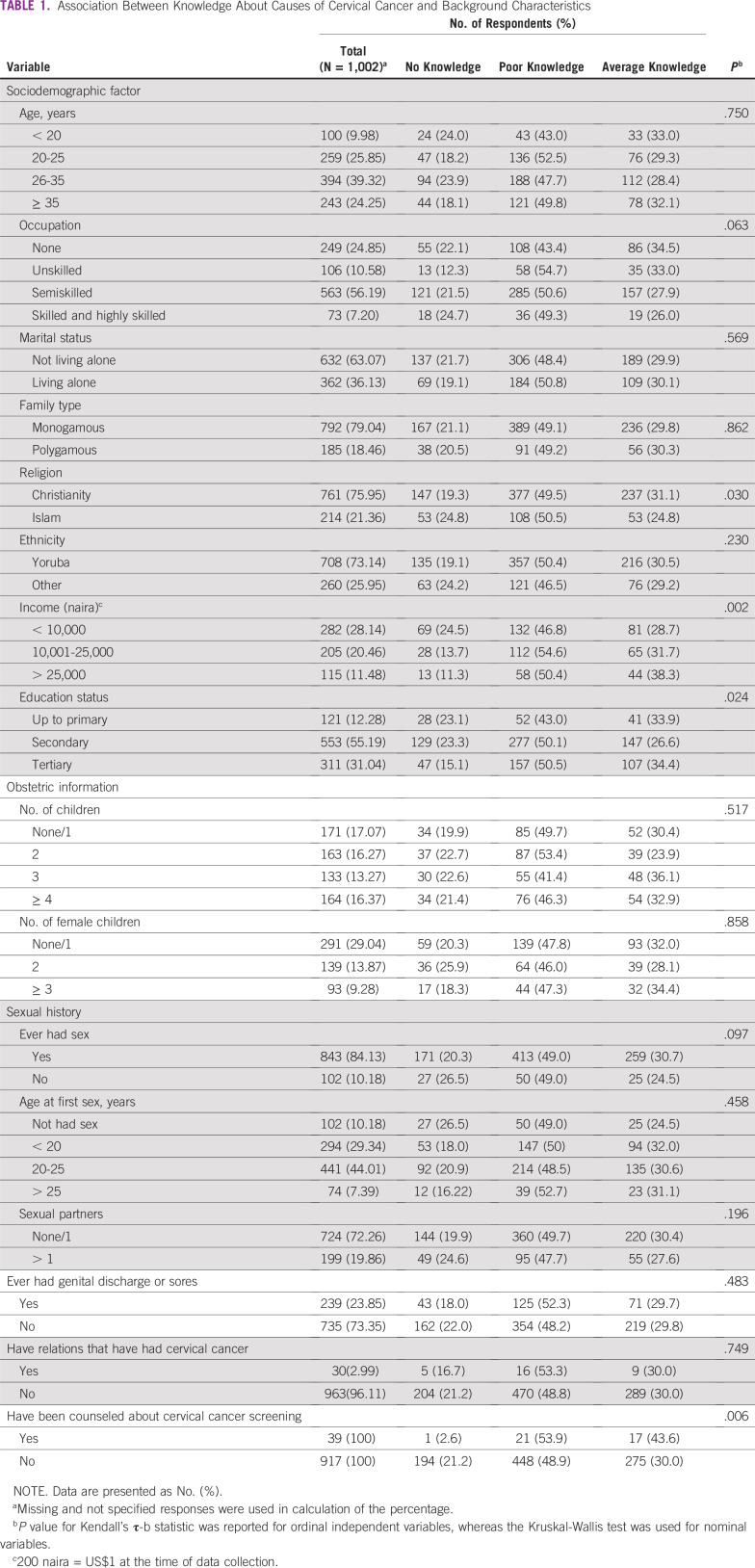
Association Between Knowledge About Causes of Cervical Cancer and Background Characteristics

### Assessment of Respondents’ Knowledge About Causes of Cervical Cancer

The most common correct risk factor for cervical cancer that respondents selected was history of multiple sexual partners (52.2%), followed by engagement in unprotected vaginal sex (48.4%), history of sexually transmitted infection (46.4%), smoking (41.5%), and HPV (40.8%; Table 2). The majority of respondents had a poor knowledge score for causes of cervical cancer. The median correct knowledge score for causes of cervical cancer was 3 (IQR, 0-4). Two hundred ninety-nine respondents (29.8%) had a knowledge score of ≥ 5, and only 1 of these respondents had a score of 7. Approximately 27.4% of respondents had a knowledge score of 0 regarding causes of cervical cancer.

**TABLE 2:**
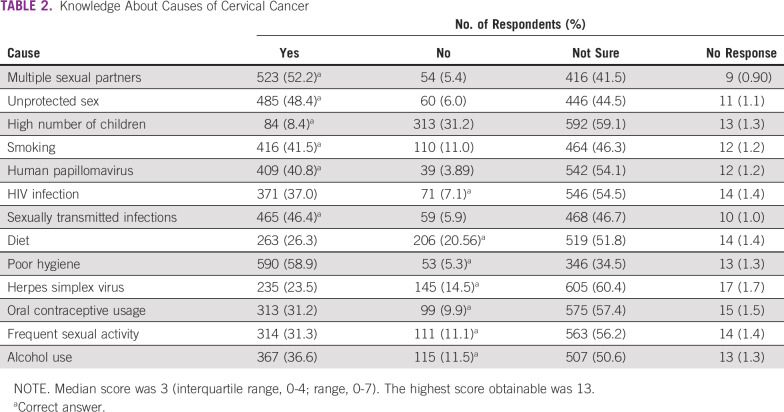
Knowledge About Causes of Cervical Cancer

### Assessment of Respondents’ Knowledge About Strategies to Prevent Cervical Cancer

More than half of respondents selected HPV vaccination (59.8%), inspection of the cervix (56.3%), and abstinence from sexual intercourse (54.0%) as correct strategies to prevent cervical cancer (Table 3). Respondents’ median correct knowledge score regarding strategies to prevent cervical cancer was 3 (IQR, 0-5), and 26.3% of respondents had a knowledge score of 0. Two hundred seventy-four respondents (27.4%) had a correct knowledge score of ≥ 5. One in 10 respondents (10.2%) had a correct knowledge score of 6.

**TABLE 3:**
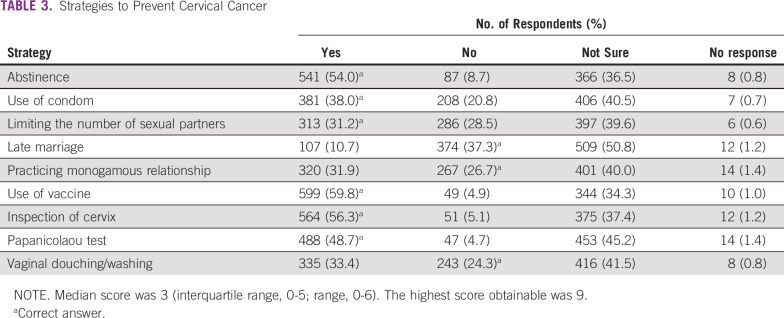
Strategies to Prevent Cervical Cancer

### Knowledge About Causes of Cervical Cancer and Associated Factors

Table 1 lists the associations between respondents’ knowledge regarding causes of cervical cancer and the selected response variables. A significant proportion of respondents who were Christians (*P* = .030) had better knowledge regarding cervical cancer causes than respondents of other religions. In addition, income (*P* = .002), occupation (*P* = .063), educational status (*P* = .024), ever having had sex (*P* = .097), and previous counseling about cervical cancer screening (*P* = .006) were associated with knowledge about cervical cancer causes.

In Table 4, the ordinal logistic regression of factors associated with knowledge about causes of cervical cancer is shown. In model 3, Muslim religion (adjusted odds ratio [AOR], 0.61; 95% CI, 0.40 to 0.92), history of multiple sexual partners (AOR, 0.64; 95% CI, 0.43 to 0.95), and no previous experience of being counseled about cervical cancer screening (AOR, 0.34; 95% CI, 0.15 to 0.80) were associated with a lower odds of being knowledgeable about causes of cervical cancer. Respondents with an income > 25,000 naira (odds ratio, 1.60; 95% CI, 1.01 to 2.56) had higher odds of being knowledgeable about causes of cervical cancer. For models 1 and 2, insignificant Brant tests and information criteria (AIC and BIC) agreed that the parallel lines assumptions were not violated and the ordered logistic regression was more parsimonious. However, model 3 showed a significant Brant test (*P* = < .001), which suggested that there was a violation of the parallel lines assumption, and the partial proportional odds models fitted were the same as those estimated by the ordered logistic regression. The AIC (1,041.14) and BIC (1,092.19) suggest that the ordinary logistic regression model 3 appears to be more parsimonious, and it was an improvement over models 1 and 2.

**TABLE 4:**
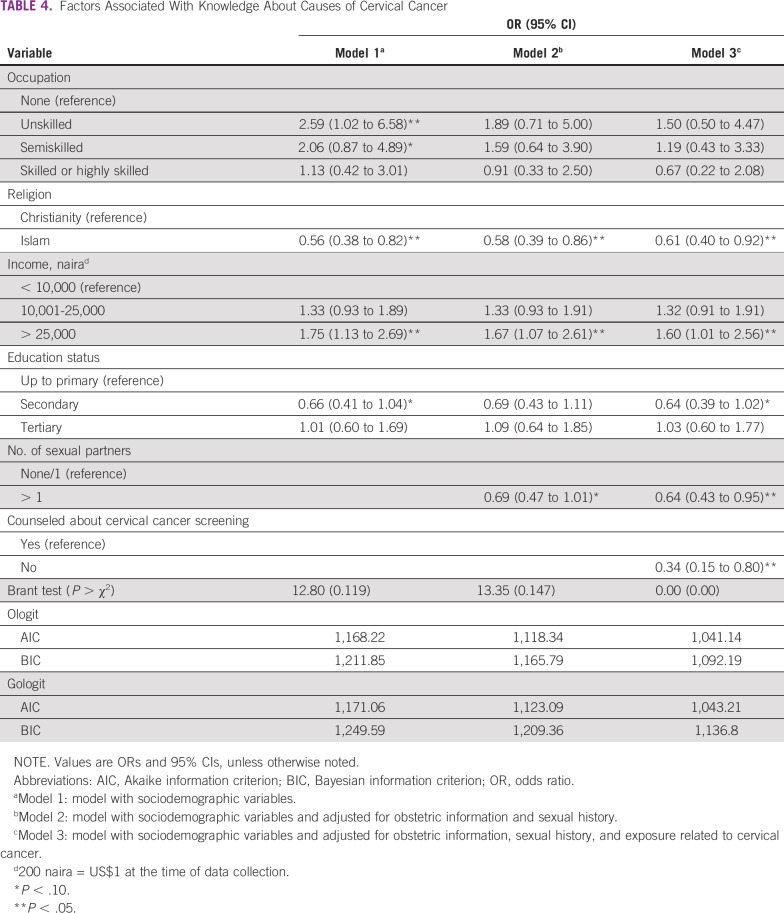
Factors Associated With Knowledge About Causes of Cervical Cancer

### Knowledge About Prevention of Cervical Cancer and Associated Factors

Table 5 lists the associations between respondents’ knowledge about prevention of cervical cancer and the selected response variables. A significant proportion of respondents who were Yoruba (*P* = .020), compared with those of other ethnic groups, and who were sexually active (*P* = .096), compared with those with no previous sexual experience, had better knowledge of cervical cancer causes. In addition, income (*P* = .001), multiple sexual partners (*P* = .053), and previous counseling about cervical cancer screening (*P* = .037) were found to be associated with knowledge about prevention of cervical cancer.

**TABLE 5:**
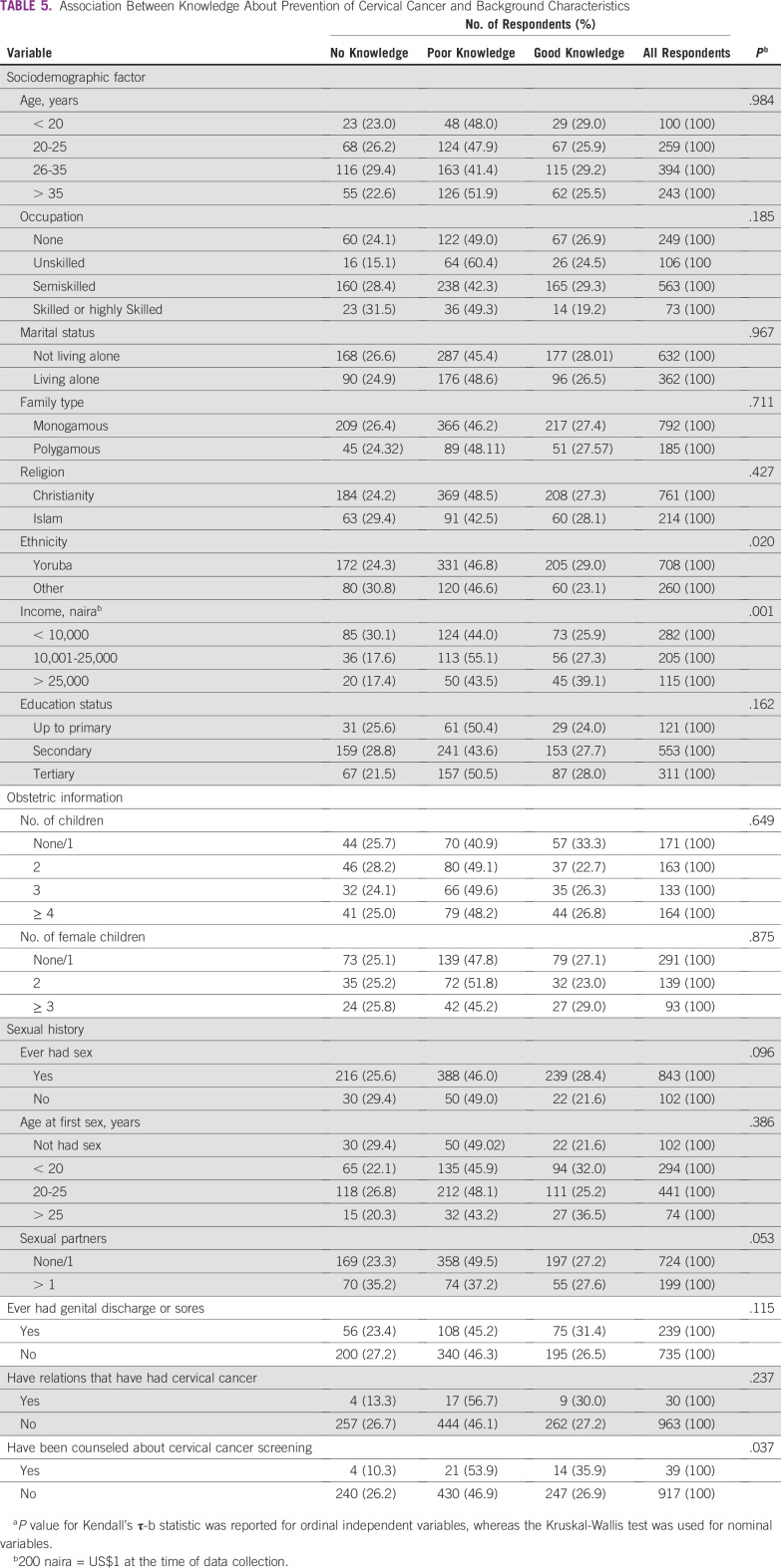
Association Between Knowledge About Prevention of Cervical Cancer and Background Characteristics

In Table 6, the partial proportional odds model of factors associated with knowledge about prevention of cervical cancer was fitted. The model consists of 2 panels (poor knowledge and good knowledge), as opposed to ordered logistic regression used in Table 6. In model 3, respondents belonging to ethnic groups other than Yoruba (AOR, 0.60; 95% CI, 0.40 to 0.90) and respondents with multiple sexual partners (AOR, 0.52; 95% CI, 0.33 to 0.83), compared with those with ≤ 1 sexual partner, had lower odds of having sufficient knowledge regarding strategies to prevent cervical cancer after adjusting for counseling about cervical cancer screening. In contrast, respondents who were unskilled workers (AOR, 3.19; 95% CI, 1.08 to 9.40), compared with those with no paid job, and respondents with income > 25,000 naira (AOR, 1.94; 95% CI, 1.21 to 3.12), compared with those with income < 1,000 naira, had higher odds of having good knowledge regarding strategies to prevent cervical cancer after adjusting for counseling about cervical cancer screening. The AIC and BIC suggest that model 2 is an improvement over model 1 and also suggest that model 3 (AIC, 1,060.97; BIC, 1,129.18) is of best fit.

**TABLE 6:**
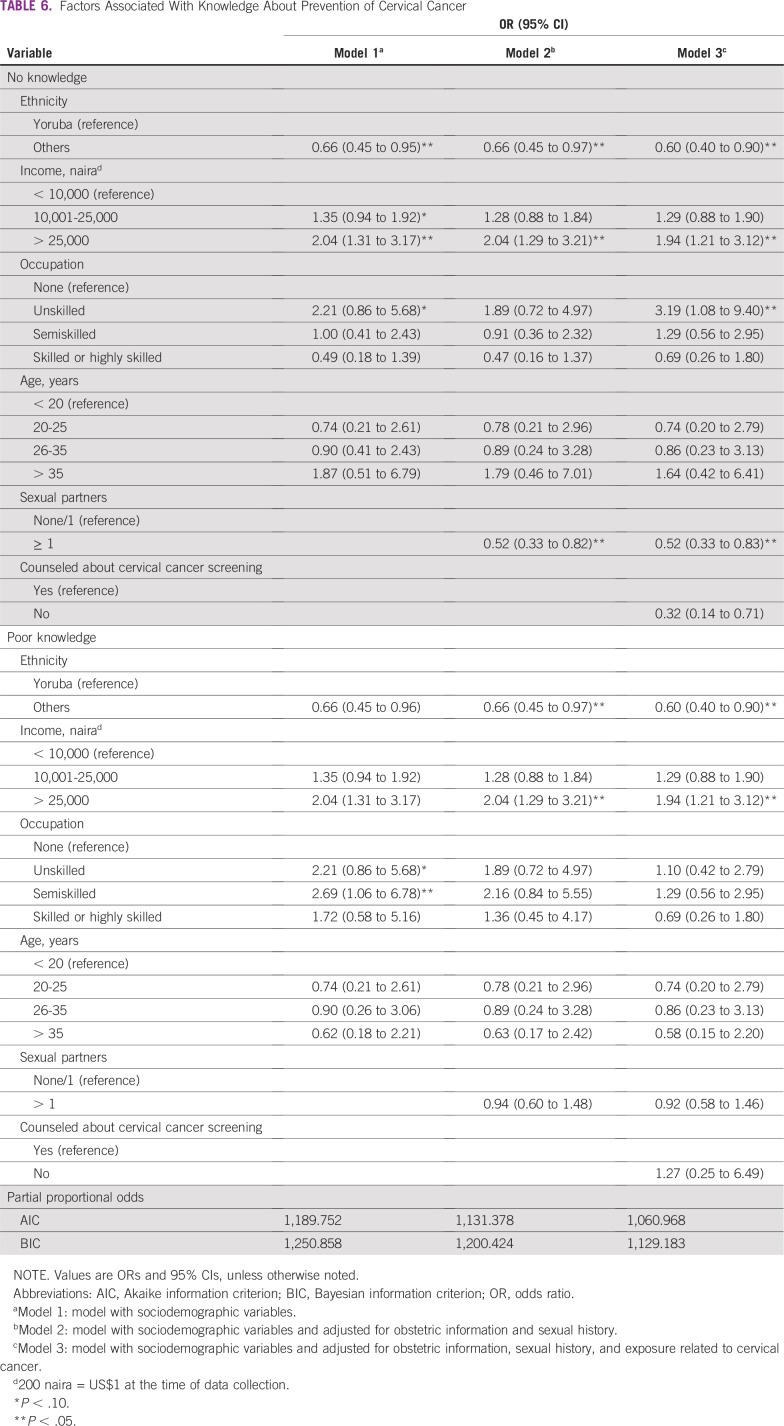
Factors Associated With Knowledge About Prevention of Cervical Cancer

## DISCUSSION

We used data collected from a community in Ibadan to demonstrate that women age 18-49 years in Nigeria had poor knowledge of risk factors and prevention strategies associated with cervical cancer. The knowledge of risk factors of cervical cancer was found to be associated with income, religion, history of multiple sexual partners, and lack of ever receiving counseling on cervical cancer. Specifically, women who had high-income jobs tended to have better knowledge of risk factors associated with cervical cancer. In contrast, Islamic women, women who reported a history of multiple sexual partners, and women who had never been counseled on cervical cancer had poor knowledge of risk factors associated with cervical cancer.

In this study, knowledge of preventive strategies for cervical cancer was associated with ethnicity, multiple sexual partners, income, and occupation. Women who were of non-Yoruba ethnic group and who reported multiple sexual partners had poor knowledge regarding prevention of cervical cancer. However, good knowledge of prevention of cervical cancer was associated with being a high-income earner or unskilled worker, compared with not having a job. It is plausible that the high proportion of unskilled women interviewed in this study had at least secondary education, and they could have had access to other sources of information, such as radio or television and social media. A study among Zimbabwean women age ≥ 25 years found poor knowledge about cervical cancer among those with no household income or those with income with < US$600, compared with women with higher household income.^24^

The poor knowledge of risk factors associated with cervical cancer among women observed in this study has been previously reported among women in other communities in Nigeria and other countries in sub-Saharan Africa.^12,25-27^ However, 2 Nigerian studies reported high knowledge among women who were secondary school teachers and health care providers. Expectedly, a few studies have also shown high knowledge of cervical cancer among 79.0% to 100.0% of women who had been counseled or previously exposed to health education sessions on reproductive health, including cancers.^16,27,28^ In this study, the only risk factor that more than half of participants knew correctly was history of multiple sexual partners. Less than half of respondents knew that unprotected sex, sexually transmitted infection, smoking, and HPV infection are risk factors for cervical cancer. It is plausible that a high proportion of participants mentioned multiple sexual partners as a risk factor of cervical cancer because of the general belief in some Nigerian communities that cervical cancer is a disease associated with promiscuity.^29^ For example, most women in a qualitative study that was conducted in a community in Lagos, Nigeria, believed that cervical cancer is a direct effect of promiscuity.^30^ However, these participants did not provide further information on the relationship between unprotected sex, sexually transmitted infection, and HPV infection and the risk of cervical cancer.^30^

Generally, knowledge about prevention strategies was poor. In this study, knowledge of participants about the primary prevention of cervical cancer appeared to be greater than knowledge regarding secondary prevention strategies. Administration of HPV vaccine and abstinence from sexual activity were the 2 most common primary prevention strategies selected by the participants. The choice of abstinence from sexual activity as a prevention strategy may reinforce the belief that cervical cancer is associated with sex. It is imperative to investigate further the connection between beliefs regarding cervical cancer and sex among women in Nigeria. The association with sexual activity alone or promiscuity might be difficult to justify as correct knowledge.

In this study, a significant number of participants had misconceptions about the risk factors associated with cervical cancer. The most common misconception was the association of poor hygiene with cervical cancer. Some of these misconceptions had been previously reported, including among health care workers.^31^ A Zambian study found that some of these misconceptions were related to individual and community perceptions of the disease.^32^ Some previously documented misconceptions about risk factors for cervical cancers include heredity, familial factors, insertion of herbs into private parts, witchcraft or satanic causes, and association with sex, among others.^32^

This study has some limitations. The cross-sectional design of the study makes it difficult to draw causality between factors associated with knowledge of risk factors and preventive strategies of cervical cancer. The interpretation of participants’ understanding of the question was based on the assumption that the interviewers correctly explained the meaning of cervical cancer to each participant. In Mokola, Nigeria, women generally access health care services at the primary health care centers and private hospitals for basic health needs and at public secondary health facilities and University College Hospital for specialized services. Although participants were not asked about where they specifically would seek care for cervical cancer screening in this study, our interaction with officials of Ibadan North Local Government indicated that cancer screening services are not available within the Mokola community. Rather, only women who are referred to the specialist or teaching hospitals usually have the opportunity for cervical cancer screening.

Despite these potential limitations, this study used a robust technique to assess risk factors associated with knowledge of participants, and we interpreted our results based on the model with the best fit. The study provided 2 levels of domain knowledge—risk factors/causes and prevention—that are important in promoting health awareness about cervical cancer at the population level.

This study demonstrates that Nigerian women lack adequate knowledge about risk factors and preventive strategies for cervical cancer. Women with high socioeconomic status tend to have better knowledge about cervical cancer compared with those from the lowest socioeconomic class. We recommend that future studies include interventions that will promote better knowledge among women and other people in the community. This is probably the first step to ameliorate the burden of cervical cancer in Nigeria. We recommend investment in innovative community mobilization to educate women on the risk factors associated with cervical cancer and prevention strategies. It is also imperative that women are counseled on the role of HPV vaccination as a primary prevention and screening for premalignant lesions as secondary prevention of cervical cancer.
